# Magnesium Salt, a Simple Strategy to Improve Methadone Analgesia in Chronic Pain: An Isobolographic Preclinical Study in Neuropathic Mice

**DOI:** 10.3389/fphar.2020.00566

**Published:** 2020-05-08

**Authors:** Valeria González, Teresa Pelissier, Victoria Cazanga, Alejandro Hernández, Luis Constandil

**Affiliations:** ^1^Laboratory of Neurobiology, Department of Biology, Faculty of Chemistry and Biology, University of Santiago of Chile, Santiago, Chile; ^2^Center for the Development of Nanoscience and Nanotechnology (CEDENNA), Santiago, Chile

**Keywords:** neuropathic pain, magnesium, isobolographic study, methadone, pain treatment

## Abstract

Analgesic efficacy of methadone in cancer and chronic non-cancer pains is greater than that of other opioids, probably because of its unique pharmacokinetics properties and also because it targets glutamatergic receptors in addition to µ-opioid receptors. However, methadone has drawbacks which are clearly related to dosing and treatment duration. The authors hypothesized that the antinociceptive efficacy of methadone could be synergistically potentiated by magnesium and copper salts in a preclinical mouse model of chronic pain, using the intraplantar formalin test as algesimetric tool. The spared nerve injury mice model was used to generate mononeuropathy. A low dose (0.25%) formalin was injected in the neuropathic limb in order to give rise only to Phase I response, resulting from direct activation by formalin of nociceptive primary afferents. Licking/biting of the formalin-injected limb was evaluated as nociceptive behavior during a 35-min observation period. Dose-response curves for intraperitoneal magnesium sulfate (10, 30, 100, and 300 mg/kg i.p.), copper sulfate (0.1, 0.3, 1, and 3 mg/kg i.p.) and methadone (0.1, 0.3, 1, and 3 mg/kg i.p.) allowed to combine them in equieffective doses and to determine their interaction by isobolographic analysis. Magnesium sulfate, copper sulfate and methadone dose-dependently decreased the nociceptive response evoked by formalin injection, the respective ED_50_ being 76.38, 1.18, and 0.50 mg/kg i.p. Isobolographic analysis showed a superadditive interaction for magnesium and methadone. Indeed, despite that both ED_50_ are obviously equieffective, the ED_50_ for the MgSO4/methadone combination contained less than one third of the methadone having the ED_50_ for methadone alone. For the CuSO_4_/methadone combination, the interaction was only additive. Extrapolated to clinical settings, the results suggest that magnesium salts might be used to improve synergistically the efficacy of methadone in neuropathy, which would allow to reduce the dose of methadone and its associated side effects.

## Introduction

Among the variety of opioid drugs currently used to manage cancer and chronic non-cancer pains, methadone is well positioned because: (i) methadone has no known active metabolites, it is well absorbed by oral and rectal routes, suffers less first pass metabolization and has a lesser interindividual variation in bioavailability than oral morphine (Gourlay et al., 1986); (ii) analgesic efficacy during chronic dosing is greater (Davis and Walsh, 2001) and opioid escalation is lesser (Mercadante et al., 1998) in patients treated with methadone than those treated with morphine; (iii) methadone displays antagonistic properties at the *N-*methyl-D-aspartate (NMDA) receptor (Ebert et al., 1995; Gorman et al., 1997), which is known to be involved in chronic pain; and (iv) methadone acts as an inhibitor of 5-hydroxytryptamine and norepinephrine uptake (Codd et al., 1995), a mechanism classically associated to pain control by tricyclic antidepressants, particularly important in the case of neuropathic pain.

Nevertheless, besides to those adverse effects that are common for all opioids (i.e., addiction, sedation, nausea, and respiratory depression), methadone has some drawbacks. First, its long and variable half-life can lead to accumulation and associated side effects in some patients, such as respiratory arrest in patients without prior opioid treatment and in those with a history of sleep apnea, severe asthma or respiratory failure (Bruera and Sweeney, 2002; Brown et al., 2004). Second, methadone interacts with other drugs that inhibit or activate the cytochrome P450 system, which is involved in the methadone metabolism (Iribarne et al., 1996; Herrlin et al., 2000). Third, weight gain (Dyer and White, 1997) and sexual dysfunction (Spring et al., 1992) are commonly reported among patients on methadone maintenance. Although these drawbacks may discourage the use of methadone for chronic non-cancer pain, therapeutic methadone use has increased 167.0% from 2000 to 2014 globally and 205.2% in the United States (Manchikanti et al., 2018). The drawbacks of chronic methadone administration are clearly related to dosing and treatment duration both for cancer (McPherson et al., 2018) and non-cancer (Els et al., 2017) pain. Consequently, the possibility of enhancing the analgesic effect of methadone (and therefore of reducing its dose) by combining it with other non-opioid antinociceptive drugs could significantly help to reduce the side effects and risks associated with the therapeutic use of this drug. Combination of methadone with tricyclic antidepressants (Banks et al., 2010; Schreiber et al., 2014), methylphenidate (Schreiber et al., 2017), delta9-tetrahydrocannabinol (Cichewicz et al., 1999) and ketamine (Pelissier et al., 2003) have already been reported, but almost in acute preclinical pain models. Methadone/ketamine (de Godoy et al., 2013) and methadone/ibuprofen (Ferrer-Brechner and Ganz, 1984) combinations have been used as antinociceptive agents in the clinic, but controlled studies reported that ketamine alone is more effective than the methadone/ketamine combination (Rigo et al., 2017) and that at the long-term nonsteroidal anti-inflammatory drugs (NSAIDs) can result in increased risk of gastrointestinal and cardiovascular side-effects (Wehling, 2014; Ho et al., 2018).

Here, we propose to study whether the antinociceptive efficacy of methadone could be potentiated by magnesium and copper salts in a preclinical model of chronic pain. Magnesium ions are coactivators of the activity of many enzymes and regulate the conductance of the NMDA receptor channel in the central nervous system (Swaminathan, 2003; Seo and Park, 2008), which play a crucial role in the mechanisms of chronic pain. Clinical trials showed that systemic Mg^2+^, used as adjuvant medication of opioids (mostly morphine, but also fentanyl and tramadol), significantly reduced opioid consumption in acute intraoperative and post-operative pain complaints (Bujalska-Zadrożny et al., 2017), but no similar data exist regarding chronic pain syndromes (Kreutzwiser and Tawfic, 2019). On the other hand, copper has demonstrated antinociceptive properties against various pain modalities in preclinical studies hot plate, tail flick tests, and in the writhing test (Tamba et al., 2013), formalin test (Cazanga et al., 2018), adjuvant arthritic rat pain model (Okuyama et al., 1987), and that it may enhance the peripheral analgesic effect of fenoprofen (Gumilar et al., 2012)) and the central analgesic effect of ketamine (Cazanga et al., 2018), but copper has not yet been tested as antinociceptive agent in humans *via* systemic route.

Thus, the aim of the present study was to evaluate the antinociceptive effect of magnesium and copper salts in a neuropathic mice model using the intraplantar formalin test, and to examine whether their effects may interact synergistically with methadone-induced antinociception using isobolographic analysis.

## Materials and Methods

### Animals

Naïve outbred CF1 male adult mice weighing 28–33 g were used for the study. Animals were housed 6 per cage and maintained with controlled temperature (21 ± 1°C) and light conditions (12:12 h light-dark cycle, lights on at 7:00 am). In total, one hundred and five mice were used in the experiments. Animals had *ad libitum* access to food and water and were allowed to habituate to the housing facility for one week before the beginning of experiments. The experimental procedure was achieved during the light phase, between 9:00 am and 12:00 am, in a quiet room. The housing conditions and experimental procedures were approved by the Bioethics Committee of the University of Santiago de Chile, and were in agreement with the ethical guidelines published by the International Association for the Study of Pain and with the Guide for the Care and Use of Laboratory Animals of NIH (National Research Council, 2011). To determine the number of required mice in each experimental group, we conducted a sample size power analysis by using the G*Power 3 Software (Faul et al., 2007). All the experimental measurements were performed in blinded condition. Each mouse was sacrificed at the end of the experiment by a carbon dioxide overdose.

### Neuropathy

Neuropathy was induced by using the spared nerve injury mice model proposed by Omori et al. (2009), which is a modification of the spared nerve injury rat model described by Decosterd and Woolf (2000) resulting in early, prolonged, and robust changes in mechanical sensitivity and thermal responsiveness that closely mimic many features of clinical neuropathic pain. In the original rat model of Decosterd and Woolf (2000), two of the three terminal distal branches of the sciatic nerve were axotomized (the tibial and common peroneal nerves), sparing only one (the sural nerve), whereas in the present mice version of the model only the sural nerve was transected, sparing the tibial and common peroneal nerves. Since the sural nerve contains almost no motor fibers (Peyronnard and Charron, 1982; Schmalbruch, 1986), this procedure allowed to generate a neuropathic pain model in mice (Omori et al., 2009) and rats (Bravo et al., 2014) in which the posture and motor functions of the hindpaw are preserved, without affecting the evaluation of pain-like responses of the paw. Briefly, animals were anesthetized with 400 mg/kg i.p. of 7% chloral hydrate solution (w/v) and a skin incision approximately 10 mm long was made in the right hindpaw at the level of sciatic nerve. The subcutaneous tissue was dissected, and the biceps femoris muscle was freed from the pelvic and vertebral heads to expose the sciatic nerve. The nerve path was then followed until its split into three branches: the sural, common peroneal, and tibial nerves. The sural nerve was cut 2 mm from its emergence, and the overlying tissues were sutured in layers. During the following 2 days after surgery, animals were daily given 3 mg/kg s.c. of the analgesic ketoprofen and 5 mg/kg s.c. of the antimicrobial agent enrofloxacin. The neural lesion described above resulted in thermal hyperalgesia of the mouse hindpaw, as measured in the hot-plate test, that persisted for at least 28 days (data not shown).

### Drugs

Magnesium sulfate heptahydrate (Fresenius Kabi, Santiago, Chile) and copper sulfate pentahydrate (Winkler, Santiago, Chile) were dissolved in physiological saline (0.9% NaCl) and administered *via* i.p. route, in a volume 0.5 ml. Doses of magnesium sulfate were 10, 30, 100, and 300 mg/kg (four groups of five mice each) and doses of copper sulfate were 0.1, 0.3, 1, and 3 mg/kg (four groups of five mice each). Methadone chlorhydrate (Laboratorio Biosano, Santiago, Chile) was administered i.p. (0.5 ml) at doses of 0.1, 0.3, 1, and 3 mg/kg (four groups of five mice each). Controls groups (five mice) received 0.5 ml of 0.9% NaCl. Thus, each mouse was given only one injection of a determined drug dose or of the solvent used.

### Behavioral Assessment: Formalin Test

The intraplantar formalin test was chosen instead mechanical or thermal pain testing, because these later are mostly based on evoked withdrawal responses that do not measure pain itself but the threshold of hyperactive reflexes that accompany pain. To run the formalin test in mice with spared nerve injury, it was used a lower formalin concentration (0.25%) than those regularly utilized for formalin testing in healthy mice (2 to 5%). This low formalin concentration gives rise only to Phase I response (direct activation by formalin of the transient receptor potential ankyrin 1, TRPA1, existing in nociceptive primary afferents) but not to Phase II response (secondary activation of nociceptive primary afferents by molecules released by neighboring injured cells, *via* formalin covalent crosslinks to proteins that disrupt cells membranes) when applied to neuropathic mice (see below in *Results*, and also see Abe et al., 2011), thereby minimizing interaction during pain testing with the neurogenic inflammation process that occurs in most models of experimental neuropathy with peripheral lesion (Meacham et al., 2017). Therefore, with this paradigm of low formalin concentration, the pain response is mostly due to direct nociceptor stimulation, as occurs with mechanical and thermal nociceptive stimuli, but with the advantage of measuring the pain response itself to nociceptor activation and not merely the threshold for eliciting a pain response as usually occurs with mechanical and thermal testing.

The animals were acclimatized in the experimental room 2 h before beginning of experiments. Fifteen min before the behavioral evaluation, mice were given a single injection either of saline (controls), MgSO_4_ alone, CuSO_4_ alone, methadone alone, MgSO_4_ plus methadone, or CuSO_4_ plus methadone. Behavioral testing was carried out by a researcher who was blind to the particular drug treatment given to each animal. For this, mice were situated into an acrylic cylinder (25 cm high x 25 cm in diameter) enclosed by two mirrors placed perpendicularly to each other. Previous to testing, each mouse was positioned into the cylinder for 10 min to acclimatize and minimize stress. Mice were then gently restrained and 20 μl of 0.25% formalin solution were injected either into the plantar surface of the right hindlimb. The intraplantar formalin test was performed as described by Cazanga et al. (2018). The nociceptive behavior evaluated was the licking/biting of the injected limb and the test was run during a 35-min observation period starting from the time of formalin administration, which was divided into seven blocks of 5-min each. A nociceptive score was determined for each block by measuring the number of seconds that the animals spent the nociceptive behavior (licking/biting the formalin-injected limb).

**Figure 1:**
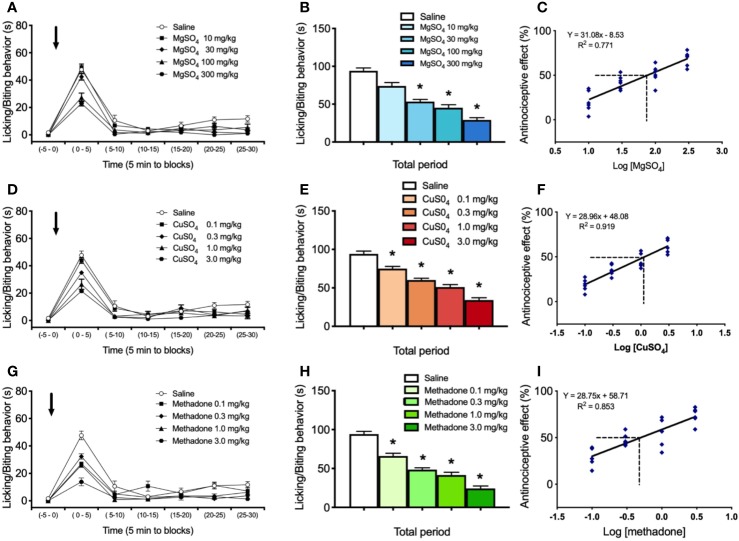
Effect of i.p. administration of saline, MgSO_4_, CuSO_4_, or methadone on licking/biting behavior elicited by intraplantar administration of 0.25% formalin in the neuropathic hindlimb of mice. Saline, MgSO_4_, CuSO_4_, or methadone were administered as a single i.p. injection 15 min before intraplantar formalin administration. **(A)** Time-course of effects of saline and 10, 30, 100, and 300 mg/kg of MgSO_4_ in nociceptive response, expressed as seconds spending licking/biting activity. **(B)** Global nociceptive score (∑NS) of licking/biting behavior for Total time of observation in intraplantar formalin test after i.p. administration of saline or increasing doses of MgSO_4_. **(C)** Dose-response data representing the antinociceptive effect (%) of MgSO_4_, expressed as dose logarithm. The ED_50_ was calculated from the regression line and is shown with segmented line. **(D)** Time-course of effects of saline and 0.1, 0.3, 1, and 3 mg/kg of CuSO_4_ in nociceptive response, expressed as seconds spending licking/biting activity. **(E)** Global nociceptive score (∑NS) of licking/biting behavior for Total time of observation in intraplantar formalin test after i.p. administration of saline or increasing doses of CuSO_4_. **(F)** Dose-response data representing the antinociceptive effect (%) of CuSO_4_, expressed as dose logarithm. The ED_50_ was calculated from the regression line and is shown with segmented line. **(G)** Time-course of effects of saline and 0.1, 0.3, 1, and 3 mg/kg of methadone in nociceptive response, expressed as seconds spending licking/biting activity. **(H)** Global nociceptive score (∑NS) of licking/biting behavior for Total time of observation in intraplantar formalin test after i.p. administration of saline or increasing doses of methadone. **(I)** Dose-response data representing the antinociceptive effect (%) of methadone, expressed as dose logarithm. The ED_50_ was calculated from the regression line and is shown with segmented line. For **(A, D, G)**: Arrows indicate formalin injection. For **(B, E, H)**: Each bar represents the mean ± SEM of 5 independent determinations. Intergroup statistics were compared by One-way ANOVA followed by Bonferroni’s multiple comparison *post hoc* test (*p < 0.01). For **(C, F, I)**: The equation for linear regression and the goodness of fit (R^2^) are shown in each graph.

**Figure 2:**
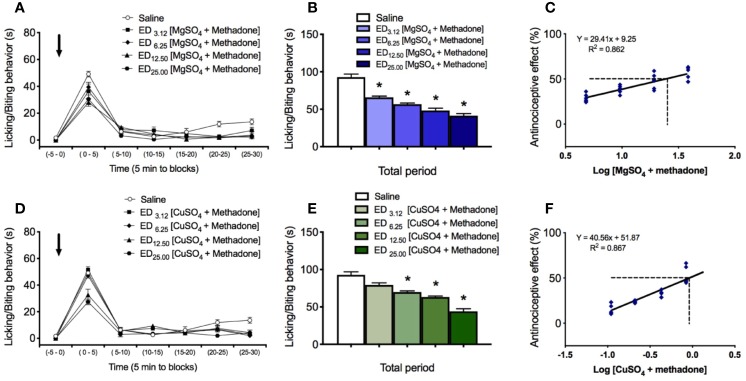
Effect of i.p. administration of saline and of the combinations of either magnesium sulfate or copper sulfate with methadone, on licking/biting behavior elicited by intraplantar administration of 0.25% formalin in the neuropathic hindlimb of mice. Saline and combinations MgSO_4_/methadone or CuSO_4_/methadone, were administered as a single i.p. injection 15 min before intraplantar formalin administration. **(A)** Time course of effects of saline and MgSO_4_/methadone combinations administered in fixed ratios of 1/2, 1/4, 1/8, and 1/16 of their respective ED_50_, expressed as seconds spending licking/biting activity. **(B)** Global nociceptive score (∑NS) of licking/biting behavior for Total time of observation in intraplantar formalin test after i.p. administration of saline or increasing equieffective doses of MgSO_4_/ketamine combination. **(C)** Dose-response data representing the antinociceptive effect (%) of MgSO_4_/methadone combination, expressed as dose logarithm. The respective ED_50_ were calculated from the regression lines and are shown in each figure with segmented line. **(D)** Time course of effects of saline and CuSO_4_/methadone combinations administered in fixed ratios of 1/2, 1/4, 1/8, and 1/16 of their respective ED_50_, expressed as seconds spending licking/biting activity. **(E)** Global nociceptive score (∑NS) of licking/biting behavior for Total time of observation in intraplantar formalin test after i.p. administration of saline or increasing equieffective doses of CuSO_4_/ketamine combination. **(F)** Dose-response data representing the antinociceptive effect (%) of CuSO_4_/methadone combination, expressed as dose logarithm. The respective ED_50_ were calculated from the regression lines and are shown in each figure with segmented line. For **(A, D)**: Arrows indicate formalin injection. For **(B, E)**: Each bar represents the mean ± SEM of five independent determinations. Intergroup statistics were compared by One-way ANOVA followed by Bonferroni’s multiple comparison *post hoc* test (*p < 0.01). For **(C, F)**: The equation for linear regression and the goodness of fit (R^2^) are shown in each graph.

As it is known (e.g., Zhao et al., 2003), the time course of the nociceptive response to formalin is usually studied by plotting the individual nociceptive scores obtained during the first 10 min following intraplantar formalin injection (the so-called Phase I) and between 10 and 35 min after formalin (the so-called Phase II). Since no Phase II response could be observed with the intraplantar injection of 0.25% formalin (see the time-course of nociceptive scores in **Figures 1** and **2**), in the present study the data was analyzed as compiled for the 35-min total time of observation. By summating the seven individual nociceptive scores (NS) recorded during the total time of observation, a global nociceptive score (∑NS) was obtained. This was subsequently used to calculate the antinociceptive effect of each dose of drug, as:

Antinociceptive effect (%)=[(∑NSsaline−∑NSdrug)/∑NSsaline]×100

where ∑NSsaline is the algebraic sum of the scores under saline and ∑NSdrug is the algebraic sum of the scores under drug. Plotting the Antinociceptive effect (%) against log dose allowed for obtaining the ED_50_ (effective dose that produce the 50% of the maximal effect) by linear regression analysis.

### Isobolographic Analysis

Evaluation of the interactions of MgSO_4_ and CuSO_4_ with methadone was performed by using isobolographic analysis (Tallarida, 2000; Tallarida, 2006). The isobologram is a graphic method that involves calculating the theoretical additive dose for each level of effect and their statistical comparison with the combination dose that causes the same effect experimentally. Equieffective doses of the drugs alone are necessary to calculate the expected dose in a combination. To this end, for each drug we defined the dose that produces 50% of maximal effect (ED_50_) by using a linear regression analysis from the dose-response curve of four increasing doses of MgSO_4_, CuSO_4_, or methadone, as stated above. Once the ED_50_ of each of the three drugs was obtained, a graph was constructed by placing in the x-axis the ED_50_ of MgSO_4_ or that of CuSO_4,_ and on the y-axis the ED_50_ of methadone. The union of the two points by a straight line (isobole), also known as line of additivity, allowed establishing the expected theoretical additivity ED50 of each ion salt with methadone in the middle of the isobole. Then, a dose–response curve for the co-administration of MgSO_4_ with methadone (four groups of five mice each) or CuSO4 with methadone (four groups of five mice each) was carried out, by administering the combination in fixed ratios of 1/2, 1/4, 1/8, and 1/16 of their respective ED_50_. Each combination of each ion salt with methadone was administered as a single i.p. injection, 15 min before the intraplantar formalin injection. The relation between the experimental value (experimental ED_50_) of the combination with to the theoretical value (theoretical additivity ED_50_) determines the type of interaction: if the value is located under the line of additivity and it is statistically different from the theoretical value, the interaction is synergistic or superadditive (effect greater than the sum of the individual effects of drugs); if it is not statistically different from the theoretical value, the interaction is simple additivity (equal effect than the sum of each drug); conversely, if the experimental value is located above the line of additivity and is statistically different from the theoretical value, it is a subadditive or antagonistic interaction. This relation can be calculated by the interaction index (γ = experimental ED_50_/theoretical additive ED_50_) between the drugs tested. This index, when smaller than 1 corresponds to a synergistic interaction, when equal to 1 corresponds to an additive interaction, and when greater than 1 is an antagonistic interaction.

### Analysis of Results

The results of scores obtained were expressed as means ± S.E.M., while the computed ED_50_ values included the 95% confidence intervals. To characterize the interaction between the drugs studied, an isobolographic analysis was performed using a custom Microsoft Excel macro program based on the method described by Ronald J. Tallarida (2000; 2006; 2016), and the interaction index calculated. The results were examined using Student’s *t*-test for unpaired data. To compare the effect of the different doses of each drug or their combinations, the results were examined using one-way analysis of variance (ANOVA) followed by the Bonferroni *post hoc* multiple comparisons test. The statistical analyses were made by using Prism 7.0 Software (GraphPad Software Inc, San Diego, CA). Significance was accepted at an alpha level of 0.05.

## Results

### Antinociceptive Effect of Magnesium Sulfate, Copper Sulfate, and Methadone in Neuropathic Mice

Intraplantar administration of 0.25% formalin in the neuropathic hindpaw of mice under drug-free condition (saline controls) induced a score of nociceptive licking/biting behavior amounting to 91.9 ± 4.1 s for the total time of observation (n=5; **Figure 1B**). Administration of magnesium sulfate i.p. induced a dose-dependent reduction of the nociceptive response induced by 0.25% formalin (**Figure 1A**). Indeed, the licking/biting behavior scores amounted to 74.0 ± 5.8 s, 55.8 ± 3.7 s, 45.6 ± 4.6 s, and 32.1 ± 3.5 s, for doses of 10, 30, 100, and 300 mg/kg of MgSO_4_, respectively (n=5 for each group; **Figure 1B**), all the nociceptive scores being significantly lower to that obtained after saline administration (*p < 0.01). The calculated value of ED_50_ for MgSO_4_ was 76.38 mg/kg with a 95% confidence interval (95% CI) of 55.21 mg/kg to 105.66 mg/kg (**Figure 1C**).

Administration of copper sulfate i.p. dose-dependently reduced the formalin-induced nociceptive response (**Figure 1D**), the licking/biting behavior scores amounting to 75.1 ± 3.7 s, 59.8 ± 3.3 s, 49.6 ± 4.1 s, and 34.1 ± 3.4 s, for doses of 0.1, 0.3, 1, and 3 mg/kg of CuSO_4_, respectively (n=5 for each group; **Figure 1E**). All these nociceptive scores were significantly lower to that obtained after saline administration (*p < 0.01). The calculated ED_50_ for CuSO_4_ was 1.18 mg/kg with a 95% CI of 0.98 mg/kg to 1.42 mg/kg (**Figure 1F**).

Methadone i.p. administration induced a dose-dependent reduction of the nociceptive licking/biting scores induced by formalin administration (**Figure 1G**). Nociceptive scores were 67.4 ± 4.9 s, 49.0 ± 2.6 s, 41.2 ± 4.2 s, and 25.2 ± 3.2 s, for doses of 0.3, 1, 1, and 3 mg/kg of methadone, respectively (n=5 for each group; **Figure 1H**). All doses of methadone produced significantly lower nociceptive scores compared to saline (*p < 0.01). The ED_50_ value for methadone was 0.50 mg/kg with a 95% confidence interval (95% CI) of 0.33 mg/kg to 0.75 mg/kg (**Figure 1I**).

### Antinociceptive Effect of the Combinations of Either Magnesium Sulfate or Copper Sulfate With Methadone in Neuropathic Mice

**Figure 3:**
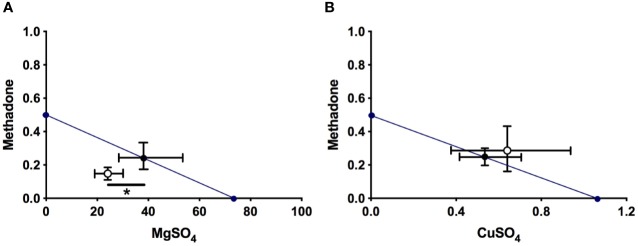
Isobolograms of interaction for MgSO_4_/methadone **(A)** and CuSO_4_/methadone **(B)** combinations in neuropathic mice, for total observation period in the intraplantar formalin test. The black circle on the straight line represents the point of theoretical additivity of the combination, whereas the white circle corresponds to the experimental point. In **(A)**: The experimental point was significantly different from the theoretical point (mean ± SEM; *p < 0.05, two-tailed Student *t* test), indicating superadditive interaction. The standard errors for MgSO_4_ and methadone are resolved into MgSO_4_ (abscissa scale) and methadone (ordinate scale) components and shown by horizontal and vertical bars, respectively. In **(B)**: The experimental point was not significantly different from the theoretical point (mean ± SEM; not significant, two-tailed Student *t* test), indicating an additive interaction of both drugs. The standard errors for CuSO_4_ and methadone are resolved into CuSO_4_ (abscissa scale) and methadone (ordinate scale) components and shown by horizontal and vertical bars, respectively.

The administration of the combination of magnesium sulfate and methadone, in equieffective proportions of their respective ED_50_, induced a dose-dependent reduction of the rubbing/scratching behavior scores in the intraplantar formalin test (**Figure 2A**). For the total period of observation after formalin injection, association of MgSO_4_ and methadone administered in fixed ratios of 1/2, 1/4, 1/8, and 1/16 of their respective ED_50_, produced nociceptive licking/biting scores of 67.1 ± 2.8 s, 58.2 ± 2.6 s, 47.6 ± 3.3 s, and 38.0 ± 3.8 s, respectively (n=5 for each group; **Figure 2B**). All these nociceptive scores were significantly lower than the obtained under saline administration (*p < 0.001). The experimental ED_50_ for the MgSO_4_/methadone association was 24.29 mg/kg (with 95% CI from 18.20 mg/kg to 32.41 mg/kg, **Figure 2C**), which can be decomposed in 24.13 mg/kg MgSO_4_ plus 0.16 mg/kg methadone. Isobolographic analysis for the administration of MgSO_4_/methadone combination showed that the experimental ED_50_ was significantly lower than the theoretical additive ED_50_ (*p* < 0.05, two-tailed Student’s *t*-test), with an interaction index γ = 0.632, which means a superadditive effect (**Figure 3A**).

The administration of the combination of copper sulfate and methadone, in fixed ratios of 1/2, 1/4, 1/8, and 1/16 of their respective ED_50_, produced nociceptive licking/biting scores of 67.1 ± 4.8 s, 58.2 ± 3.6 s, 47.6 ± 3.3 s, and 38.0 ± 4.8 s, respectively (n=5 for each group; **Figure 2E**). The three higher doses of combined copper sulfate and methadone administered led to significantly lower nociceptive scores, compared to saline administration (*p < 0.01), and the calculated experimental ED_50_ for the CuSO_4_/methadone association was 0.90 mg/kg (with 95% CI from 0.68 mg/kg to 1.32 mg/kg, **Figure 2F**), which can be decomposed in 0.63 mg/kg CuSO_4_ plus 0.27 mg/kg methadone. Isobolographic analysis for the administration of MgSO_4_/methadone combination showed that the experimental ED_50_ did not significantly differ from the theoretical additive ED_50_ (two-tailed Student’s *t*-test), with an interaction index γ = 1.148, which means simple additivity (**Figure 3B**).

## Discussion

The present results showed that both magnesium sulfate and copper sulfate produced dose-dependent antinociceptive effects in the intraplantar formalin test. This is in agreement with expectations since magnesium and copper salts have been found to induce dose-dependent antinociception in the hot-plate, tail-flick, and writhing tests (Tamba et al., 2013). Besides, the antinociceptive effects showed by magnesium and copper ions also coincide with those obtained in other studies using formalin-induced pain, where systemic magnesium improved the antinociceptive effect of ketamine (Vujović et al., 2017) and copper-NSAIDs complexes exhibited higher antinociceptive effect than NSAIDs alone (Gumilar et al., 2012). The foregoing results also showed that the MgSO_4_/methadone and CuSO_4_/methadone combinations produced higher antinociception than methadone alone, an opiate agonist that has proven to be effective in thermal and mechanical pain models (Lemberg et al., 2006), as well as in chemonociception (Miranda et al., 2014) and different models of neuropathic pain (Erichsen et al., 2005).

The main and most important result of the present study was that MgSO_4_ and methadone interacted synergistically in the intraplantar formalin pain model, which means that there was a potentiation of the antinociceptive effect of the drugs when given in combination. In fact, the ED_50_ of methadone alone was 0.50 mg/kg while the equieffective dose of the MgSO_4_/methadone combination only contained 0.16 mg/kg methadone, which means that the addition of MgSO_4_ allowed the methadone content of the dose to be reduced to less than one third. If extrapolated to humans, this might be an important finding because the side-effects of methadone in clinical settings are related to dosing and treatment duration, both for cancer (McPherson et al., 2018) and non-cancer (Els et al., 2017) pains. Indeed, according to the guidelines from the American Pain Society (Chou et al., 2014) and from the experts group of the Hospice and Palliative Care (McPherson, 2016), rotation to methadone of opioid-tolerant patients with cancer pain should be based on dose calculations, as exact opioid/methadone ratios. The same apply to chronic non-cancer pain, where the risk for addiction increases with increasing opioid doses (Huffman et al., 2015), and sleep-disordered breathing and respiratory depression may result in opioid-associated deaths demonstrating a clear relationship to dose (Walker et al., 2007; Jungquist et al., 2012).

It seems worth to remark that the superadditive interaction between MgSO_4_ and methadone, detected by isobolographic analysis upon intraplantar formalin testing, originated from parallel regression lines obtained in the dose-response plots of the individual drugs, meaning that the potency ratio for these two drugs remained constant during testing of formalin-induced pain in neuropathic rats (Tallarida, 2000; Tallarida, 2006; Tallarida, 2016). Theoretically, superadditivity in the effect of two simultaneously administered antinociceptive drugs implies that the combined molecules act on anatomically and/or functionally different substrates for nociceptive processing, which may represent different neurons, different receptors in the same neuron, or even different sites of binding in the same receptor. In this regard, it is well known that magnesium induce antinociception by antagonistic binding to NMDA receptors (Traynelis et al., 2010), while the antinociceptive effect of methadone can be explained by both agonism at µ opiate receptors and antagonism on NMDA receptors (Gorman et al., 1997).

Methadone is a potent inhibitor of [^3^H]MK-801 binding, a specific uncompetitive NMDA receptor antagonist, with a K_i_ of 0.85 ± 0.31 µM (Ebert et al., 1995). Gorman et al. (1997) reported moderate affinity, but still in the low µM range, for the displacement of [^3^H]MK-801 by *l*-, *d*-, and *dl*-methadone (K_i_ of 3.4 ± 0.3, 7.4 ± 1.2, and 8.3 ± 1.2, respectively) from non-competitive NMDA receptor sites in the rat forebrain. More recently, Matsui and Williams (2010) showed that the NMDA current induced by iontophoretic application of L-aspartate in locus coeruleus neurons was dose-dependently inhibited by *l/d* methadone with an IC50 value for *l/d*-methadone of 3.5 ± 0.3 µM, which was statistically similar to the IC50 of *d*-and *l*- methadone enantiomers. Since methadone blocked the inward but not the outward current in the NMDA channel, it can be concluded that, in addition to µ-opioid receptor binding, at low µM concentration methadone could act as a non stereoselective, uncompetitive, voltage-dependent pore blocker of the NMDA receptor. Despite that the MgSO_4_/methadone combination reported here was synergistic, it seems apparent that the effects of Mg^2+^ and methadone were redundant at the NMDA receptor, because both magnesium ions (Traynelis et al., 2010) and methadone (Gorman et al., 1997; Matsui and Williams, 2010) are open channel blockers that act as voltage-dependent uncompetitive NMDA receptor antagonists in the same site of the NMDA channel. In such a case, one drug substitutes for the other and only additivity should be expected when the two drugs are given simultaneously. Thus, it is likely that the synergy of the MgSO_4_/methadone combination reported here arose from the interaction between the blocking properties of magnesium ions in the NMDA receptor channels and the agonistic properties of methadone in µ opioid receptors. With regard to this, it has been reported that binding of opioid agonists and antagonists in brain homogenates is allosterically promoted by Mg^2+^ in a concentration-dependent manner (Rodriguez et al., 1992), which could be at the base of the synergistic effect of magnesium on methadone-induced antinociception. A rather similar synergistic interaction between methadone and ketamine in neuropathic rats has previously been reported (Pelissier et al., 2003), but the translational potential of such a combination is likely to be impaired by the well-known undesirable psychomimetic effects of ketamine (Persson, 2013). In contrast, magnesium is cheap and well tolerated by oral route, as children have been safely treated from chronic constipation with 2 ml/kg daily of milk of magnesia, i.e., 160 mg magnesium hydroxide/kg/day (Loening-Baucke and Pashankar, 2006).

As it is known, subcutaneously injected formalin into the mouse paw gives rise to phase I response by direct activation of nociceptive primary afferents *via* TRPA1 channels (McNamara et al., 2007), and to phase II response (or inflammatory phase) corresponding to a secondary activation of nociceptive primary afferents by histamine, bradykinin, cytokines, and substance P, among others mediators released by inflammatory cells (Zouikr et al., 2015). Various forms of experimental neuropathy induced by peripheral injury (Kingery et al., 1999; Botz et al., 2013; Gallo et al., 2017) have been associated to neurogenic inflammation, eliciting the release of substance P, calcitonin gene-related peptide, neurokinin A, endothelin-3, cytokines, among others cellular mediators of inflammation. Since both formalin administration and the neuropathic process upregulate rather similar inflammatory mediators, this makes difficult the interpretation of nociceptive data during phase II response. Therefore, we utilized a paradigm of low formalin concentration, as proposed by Abe et al., 2011, where only phase I pain response can be observed.

Unlike the superadditive interaction between magnesium salt and methadone, copper ions give rise only to an additive effect in the intraplantar formalin test when administered together with methadone. This different interaction, obtained through isobologram analysis of data, could be related to the already reported different mode of binding of Cu^2+^ on NMDA channels, where copper acts as a high-affinity NMDA receptor antagonist characterized by a voltage-independent mechanism of action (Herrlin et al., 2000).

Although it remains yet uncertain the mechanism underlying the ability of magnesium sulfate to exert a synergistic action upon the methadone antinociceptive effect, this issue could constitute a potential basis for future clinical applications addressed to lower methadone dosing together with a lowering of its side-effects. As pointed out elsewhere (Erichsen et al., 2005), it is not possible to determine synergism in humans due to scientific, practical and ethical reasons, and thus prior to testing drug interaction in clinical trials, studies on preclinical drug combinations should be carried out in animals to obtain the basis and rationale for further studies in humans. Among opioids, methadone should be well positioned for treat neuropathic pain because of its unique ability to target NMDA receptors in addition to its well-known effect on µ opioid receptors. However, therapeutic guidelines relegate strong opioids, including methadone, to third-line therapy in neuropathic pain mainly because of safety concerns (Finnerup et al., 2015). Drug combination strategies aimed to reduce methadone dosing—and therefore its side-effects—could be a promising therapeutic approach to optimize opioid analgesia under neuropathic pain conditions, provided the drug co-administered with methadone does not give rise to important side-effects by its own. To this end, magnesium salts probably represent the best alternative, since the European Food Safety Authority (EFSA) of the European Union states that the upper limit for magnesium (i.e., the daily dose that does not produce any observable adverse effect in healthy adult humans) is as high as 350 mg, while toxic hypermagnesemia is only seen at oral doses greater than 2500 mg daily (Scientific Committee on Food, Scientific Panel on Dietetic Products, Nutrition and Allergies, 2006). Although further studies are necessary to examine in detail the mechanism underlying the synergistic interaction between magnesium ions and methadone, it can be concluded that this association could represent a potential therapeutic strategy aimed to treat some forms of chronic pain in humans, which deserves more investigation in clinical settings.

## Data Availability Statement

The datasets generated for this study are available on request to the corresponding author.

## Ethics Statement

The animal study was reviewed and approved by Bioethics Committee of the University of Santiago de Chile.

## Author Contributions

TP and LC provided ideas or concepts for definition of intellectual context, particularly designed and performed the experiments. VG, VC, and TP performed research. AH, TP, and LC contributed new reagents/analytic tools. VG, AH, TP, and LC analyzed data. AH and LC wrote the paper. All authors of this paper have read and approved the final version of the manuscript.

## Funding

This work was supported by the Fondecyt Project (grant 1181622) and the Centers of Excellence with Basal/Conicyt financing, CEDENNA (grant AFB180001).

## Conflict of Interest

The authors declare that the research was conducted in the absence of any commercial or financial relationships that could be construed as a potential conflict of interest.
